# *Mycobacterium tuberculosis* Prolyl Oligopeptidase Induces *In vitro* Secretion of Proinflammatory Cytokines by Peritoneal Macrophages

**DOI:** 10.3389/fmicb.2017.00155

**Published:** 2017-02-07

**Authors:** Brina Portugal, Flávia N. Motta, Andre F. Correa, Diego O. Nolasco, Hugo de Almeida, Kelly G. Magalhães, Ana L. V. Atta, Francisco D. Vieira, Izabela M. D. Bastos, Jaime M. Santana

**Affiliations:** ^1^Pathogen-Host Interface Laboratory, Department of Cell Biology, The University of Brasília, BrasíliaBrazil; ^2^Faculty of Ceilândia, The University of BrasíliaBrasília, Brazil; ^3^Instituto de Patologia Tropical e Saúde Pública, Universidade Federal de GoiásGoiânia, Brazil; ^4^Physics Course and Postgraduate Program in Genomic Sciences and Biotechnology, Catholic University of BrasíliaBrasília, Brazil; ^5^Laboratory of Immunology and Inflammation, Department of Cell Biology, The University of BrasíliaBrasília, Brazil; ^6^Laboratório Central de Saúde Pública do Distrito FederalBrasília, Brazil

**Keywords:** tuberculosis, *Mycobacterium tuberculosis* protease, serine protease, prolyl oligopeptidase, proinflammatory cytokines, molecular dynamic, fluorescence spectroscopy

## Abstract

Tuberculosis (TB) is a disease that leads to death over 1 million people per year worldwide and the biological mediators of this pathology are poorly established, preventing the implementation of effective therapies to improve outcomes in TB. Host–bacterium interaction is a key step to TB establishment and the proteases produced by these microorganisms seem to facilitate bacteria invasion, migration and host immune response evasion. We presented, for the first time, the identification, biochemical characterization, molecular dynamics (MDs) and immunomodulatory properties of a prolyl oligopeptidase (POP) from *Mycobacterium tuberculosis* (POPMt). POP is a serine protease that hydrolyzes substrates with high specificity for proline residues and has already been characterized as virulence factor in infectious diseases. POPMt reveals catalytic activity upon N-Suc-Gly-Pro-Leu-Gly-Pro-AMC, a recognized POP substrate, with optimal activity at pH 7.5 and 37°C. The enzyme presents K_M_ and K_cat_/K_M_ values of 108 μM and 21.838 mM^-1^ s^-1^, respectively. MDs showed that POPMt structure is similar to that of others POPs, which consists of a cylindrical architecture divided into an α/β hydrolase catalytic domain and a β-propeller domain. Finally, POPMt was capable of triggering *in vitro* secretion of proinflammatory cytokines by peritoneal macrophages, an event dependent on POPMt intact structure. Our data suggests that POPMt may contribute to an inflammatory response during *M. tuberculosis* infection.

## Introduction

Despite global effort to stop tuberculosis (TB), it remains the second-deadliest infectious disease worldwide causing over 1 million deaths per year and an additional 0.4 million death resulting from TB disease among HIV-positive patients (WHO, 2015). The pathology results from a highly evolved and multifactorial ability of *Mycobacterium tuberculosis*, an intracellular bacterium, to prevent or evade effective host responses (Goldberg et al., 2014). The initial host response triggered against *M. tuberculosis* infection is characterized by innate immune response that involve the recruitment of inflammatory cells to the lungs followed by *M. tuberculosis* dissemination to draining lymph nodes during adaptive immune response (Chackerian et al., 2002; Reiley et al., 2008; Wolf et al., 2008). Even though *M. tuberculosis* can infect a variety of cell types, alveolar macrophages are its main niche. *M. tuberculosis* spread and dissemination is deeply correlated with its ability to infect and immunomodulate macrophages. In reaction to *M. tuberculosis* infection, macrophages upregulate effectors and signaling pathways to both prevent bacilli replication and recruit other immune cells into the site of infection (Cooper and Torrado, 2012; Sia et al., 2015). However, *M. tuberculosis* has an arsenal of potent mechanisms for evading those antimicrobial reactions, thereby changing the host immune response toward a pathological rather than a protective one. TB disease occurs when the pathological process overcomes the protective response, promoting chronic inflammation and lung damage leading to severe coughing, fever, and chest pains (Fogel, 2015). Although pulmonary TB is the most typical presentation of the disease, *M. tuberculosis* may also disseminate into a variety of organs causing extrapulmonary TB (Galimi, 2011).

*Mycobacterium tuberculosis* multidrug- (MRD) and extensively drug-resistant (XDR) strains along with HIV coinfection are recognized as predominant threats to public health and, combined to the lengthy and complex treatment for TB, they emphasize the eminent necessity to comprehend host- and pathogen-derived factors and their interactions (Roberts et al., 2013; Korb et al., 2016). Mycobacteria proteases have an active role in pathogenicity and viability of *M. tuberculosis* inside host cells (Lebrun et al., 2009; Zhao and Xie, 2011). Recently, *M. tuberculosis* zinc metalloprotease (zmp1), first known to be required for bacilli virulence and survival in macrophages (Master et al., 2008), was described to be implied with the endothelin system by cleavage of ET-1, which seems to be responsible for TB progression and inflammatory cell recruitment (Correa et al., 2014). Rv3671c (marP), an acid resistant serine-protease from *M. tuberculosis* periplasm, is responsible to maintain phagocytosed bacterial pH near neutrality in the acidic environment generated by IFN-gamma-activated macrophages. As a consequence, *M. tuberculosis* is able to resist to phagosome acidification, which is essential for *M. tuberculosis* virulence (Vandal et al., 2008; Zhao et al., 2015). With respect to proteolytic complex, *M. tuberculosis* possesses two potential ClpP proteolytic subunits (CIpP1P2) that seem to be involved in preventing the accumulation of misfolded proteins and the degradation of critical endogenous regulatory proteins. Active site mutants of CIpP1P2 showed that the enzymatic activity of each subunit is required for normal growth of *M. tuberculosis in vitro* and during infection of mice (Raju et al., 2012). Another protease already studied is Rv2224c, a cell envelope-associated predicted protease, which compromised the intracellular survival of *M. tuberculosis* into lung macrophages. Mice infected with Rv2224c mutant survived significantly longer than the wild-type infected-mice with reduced lung pathology, attenuating the virulence of *M. tuberculosis* (Lun and Bishai, 2007; Rengarajan et al., 2008; Vandal et al., 2009).

This research is focused on *M. tuberculosis* prolyl oligopeptidase (POPMt) from the serine peptidase family S9, which has not been specifically investigated in mycobacteria. Prolyl oligopeptidase (POP, EC 3.4.21.26) belongs to a specific group of enzymes that is capable of hydrolyze peptide bonds on the carboxyl side of proline residues (Koida and Walter, 1976), an unusual amino acid with a cyclic structure that confers protection to proline-containing molecules from enzymatic degradation. Alterations in human POP enzymatic activity have been detected in patients suffering from depression, mania, schizophrenia, nervous anxiety, anorexia and bulimia (Maes et al., 1995, 1998, 2001), including Alzheimer’s and Parkinson’s diseases (Mantle et al., 1996) and multiple sclerosis (Tenorio-Laranga et al., 2010). POP seems to be related to regulation of pathways involving inositol (1, 4, 5)-triphosphate (IP_3_) and it was already shown that low levels of the enzyme cause neurotrophic effects by increasing IP_3_ (Williams et al., 1999; Williams and Harwood, 2000; Maes et al., 2001). POP has also been studied as a potential therapeutic component for the treatment of celiac disease, a chronic enteropathy induced by immunotoxic and proline-rich gluten peptides (Shan et al., 2002).

Additionally, POP orthologous enzymes from some pathogens have been described as virulence factors of infectious diseases, as in the case of trypanosomiasis and schistosomiasis. In *Trypanosoma cruzi*, POPTc80 is secreted by the infective form of the parasite and hydrolyzes extracellular matrix components such as collagen and fibronectin (Santana et al., 1997). Selective and specific POPTc80 inhibitors blocked *in vitro* trypomastigotes entry into different types of non-phagocytic cells reinforcing the enzyme role in cell invasion (Vendeville et al., 1999; Joyeau et al., 2000; Grellier et al., 2001; Bastos et al., 2005). POP from *T. brucei* is active in the plasma of infected mice and is capable of hydrolyze native collagen and peptide hormones that are deregulated in sleeping sickness (Bastos et al., 2010). More recently, POP from *Schistosoma mansoni* was partially characterized and it seems to be able to cleave peptides such as vasoconstrictory angiotensin I and bradykinin. This ability may provide a survival benefit to the schistosome during its residence in and movement through the venous blood system (Fajtová et al., 2015).

In this report, we identified and characterized the biochemical activity of a recombinant POP from *M. tuberculosis*. We also produced data about its three-dimensional structure based on molecular dynamics (MDs) experiments and investigated murine macrophages immunomodulatory response elicited by POPMt.

## Materials and Methods

### Bacterial Culture and Growth Conditions

*Mycobacterium tuberculosis* H37Rv was grown at 37°C in a shaking incubator (120 rpm) in Difco^TM^ Middlebrook 7H9 broth containing 0.2% glycerol, 0.05% Tween 80, and 10% albumin-dextrose-catalase growth enrichment (Becton Dickinson and Company, Diagnostic Systems, Sparks, MD, USA) to OD_600_ of 0.5. *M. tuberculosis* H37Rv was cultured in a BSL3 facility at the Laboratório Central de Saúde Pública – Brasília – DF (Lacen – DF).

### Cloning of popmt Gene (Rv0457c)

The *popmt* gene was amplified by PCR from *M. tuberculosis* H37Rv genomic DNA using the primers: forward, 5′-AGATTACAT**ATG**ACATTTGAGCCTGCCC-3′ (*Nde* I site, underlined; initiation codon, bold) and reverse, 5′-GTTATAGGATCCT**TAG**CCGGCCAGCATCCG-3′ (*BamH* I site, underlined; stop codon, bold). The 2022 bp PCR product was digested and ligated into similarly digested pET-28a(+) (Novagen) using T4 DNA ligase (Invitrogen) in frame with N-terminus 6xHis-tag vector. The cloned *popmt* sequence was checked by DNA sequencing of both strands using T7 primers (Genscript, USA). For overexpression of his-tagged recombinant protein, the pET-28a(+):*popmt* plasmid was transformed into *Escherichia coli* BL21 (DE3) cells.

### Expression and Purification of Recombinant POPMt

Overnight culture of *E. coli* BL21 (DE3) carrying pET-28a(+):*popmt* was inoculated into 500 ml of LB broth supplemented with 30 μg/ml kanamycin and incubated at 37°C, 200 rpm until the OD_600_ reached 0.4–0.6. Standard conditions of expression were established with 0.05 mM isopropyl β-D-1-thiogalactopyranoside (IPTG) at 20°C for 16 h. Recombinant POPMt (POPMt) was purified from the soluble fraction through nickel affinity chromatography (His Bind^®^ Kit – Novagen) according to the manufacturer’s protocol. Purified POPMt was stored in 50% glycerol at -20°C. POPMt purification was analyzed on 10% SDS-PAGE followed by Coomassie Brilliant Blue R staining (Sigma-Aldrich). The protein was quantified using the molar absorption coefficient 𝜀 value of 148,865 (M^-1^cm^-1^) at 280 nm measured in water.

### Western Blot

The membrane was blocked by incubation in 5% (w/v) non-fat milk/PBS overnight at 4°C. Blot was incubated for 2 h with 1:100 POPMt diluted in 1% non-fat milk/PBS. After several washes with PBS, the membrane was incubated for 1 h with 1:1000 alkaline phosphatase-conjugated goat anti-mouse IgG (Invitrogen). Immunocomplexes were revealed with the alkaline phosphatase substrate 5-bromo-4-chloro-3-indolyl-phosphate/Nitro Blue Tetrazolium (BCIP/NBT Color Development Substrate – Promega).

### Enzymatic Characterization of POPMt

Recombinant POPMt enzymatic activity was measured using 7-amido-4-methylcoumarin (AMC). The released of AMC was monitored up to 20 min in 96-well SpectraMax M5 microplate reader (Molecular Devices) at 25°C as previously described (Grellier et al., 2001). The POPMt enzymatic activity was assayed on several AMC-containing substrates: Ala-Ala-Phe-AMC, L-Proline-AMC hidrobromide, *N*-Succinyl-Ile-Ala-AMC, *N*-Succinyl-Leu-Tyr-AMC, *N*-Succinyl-Leu-Leu-Val-Tyr-7-AMC, L-Leucine-AMC hydrochloride, Gly-Pro-AMC, *N*-Succinyl-Gly-Pro-AMC (*N*-Suc-Gly-Pro-AMC) and *N*-Succinyl-Gly-Pro-Leu-Gly-Pro-AMC (*N*-Suc-Gly-Pro-Leu-Gly-Pro-AMC), which were purchased from Sigma-Aldrich. Enzymatic reactions were performed with 25 mM Tris pH 7.5 (reaction buffer) containing 20 μM of fluorogenic substrate with or without additives (NaCl – 0 to 400 mM and DTT – 0 to 20 mM) in 100 μl final volume. The temperature assay was carried out by incubating the enzyme at different temperatures: 20, 28, 37, 40, 45, 60, or 80°C for 20 min and afterward 20 μM of substrate were added. The pH activity optimum of POPMt was determined in AMT buffer (100 mM acetic acid, 100 mM MES, and 200 mM Tris-HCl) at pHs ranging from 5.0 to 10.0 (Bastos et al., 2010). All experiments were performed in triplicate and repeated three times independently.

Kinetic parameters were determined by incubation of POPMt in reaction buffer with different concentrations (6.25–150 μM) of *N*-Suc-Gly-Pro-Leu-Gly-Pro-AMC. *K_m_* and *V_max_* were determined by hyperbolic regression according to Cornish-Bowden (Cornish-Bowden, 1976). The *k_cat_* was calculated by = V max/[E]t, where [E]_t_ is the total enzyme concentration.

Different concentrations of the inhibitors tosyl-lysylchloromethane (TLCK), *N*-*p*-Tosyl-L-phenylalanine chloromethyl ketone (TPCK), bestatin, EDTA, L-*trans*-epoxysuccinylleucylamido-(4-guanidino) butane (E-64), 1,10-phenanthroline and leupeptin (0.01–100 nM) were incubated with POPMt in 100 μl reaction buffer for 15 min at room temperature before the addition of 20 μM *N*-Suc-Gly-Pro-Leu-Gly-Pro-AMC. The enzymatic reactions were monitored as described above. The inhibition profile (IC_50_) using Z-Pro-Prolinal, a specific inhibitor of POPs, was determined by non-linear regression analysis from the residual activity versus inhibitor concentrations curve and the *Ki* values determined using the Cheng-Prusoff method K_i_= IC50/[1 + ([S]/Km)], where [S] is the concentration of the substrate (Yung-Chi and Prusoff, 1973).

### Fluorescence Spectroscopy

Fluorescence measurements were performed using an ISS K-2 (Champaign, IL, USA) spectrofluorimeter at the same temperatures used in activity assay as described above. Spectra were recorded from 305 to 450 nm using an excitation wavelength of 295 and 2 nm bandwidth for both excitation and emission. Solutions of 0.30 μM POPMt were prepared in AMT buffer at pHs ranging from 5 to 10. Measurements were carried out in a 1.0 × 1.0-cm cuvette. The final spectra were baseline corrected by subtracting buffer spectrum.

### Homology Modeling and Molecular Dynamics Simulation Parameters

The POPMt 3D-model was built based on the *Myxococcus xanthus* POP crystal structure, PDB code 2BKL (Shan et al., 2005), using the MODELLER 9v8 software (Eswar et al., 2006). Briefly, using the NCBI BLASTP webservers (Altschul et al., 1997), the POPMt sequence was submitted to a search query against a structure database [Protein Data Bank (PDB) (Berman et al., 2000)]. The PSI-BLAST algorithm was used to perform an alignment profile between all the homologous sequences and facilitate the alignment between the target sequence (POPMt) and the template. The alignment was then subjected to the MODELLER software, resulting in 5 comparative models. The one with the lowest DOPE score was selected to further refinements by MDs simulations.

The MD simulation was performed using the computational package GROMACS 4 – Groningen Machine for Chemical Simulations (Hess et al., 2008). The simulated ensemble was composed of the POPMt model immersed in 14,46 Single Point Charge (SPC) water molecules (Berendsen et al., 1981) in a cubic box with edges of 77 Å. Four sodium atoms were also included in order to neutralize the system charges. The lysine residues of the protein were protonated and an amide group was added to the C-terminus. The SETTLE algorithm was used to constrain the geometry of water molecules, and the LINCS (Hess et al., 1997) algorithm was used to constrain bond lengths. The electrostatic corrections were performed by the Particle Mesh Ewald (PME) algorithm with a cutoff radius of 1.4 nm, in order to minimize the computational time of the simulation. Derived from the Ewald summation, PME estimates in the Fourier space the long-range interactions that happen in real space (Darden et al., 1993; Essmann et al., 1995). The same radius value cutoff was also used in the van der Waals interactions. The neighbors list of each atom was updated every 10 simulation steps of 20 fs each.

The system was contained. Two steps of energy minimization were performed (2 ns each), the first used the conjugate gradient algorithm and the other one used the steepest descent algorithm. After the energy minimization step, the system went through a pressure and temperature normalization process using the integrator stochastic dynamics (SD), also for 2 ns. Thereafter, the system went through a position restrain step using the MDs integrator for another 2 ns. This ensemble was subjected to a relaxation MDs run at GridUNESP computers. The 50 ns simulation was divided into 25,000,000 steps of 2 fs each.

### Stimulation of Peritoneal Macrophages with POPMt *In vitro*

Peritoneal macrophages from naive C57BL/6 mice were harvested by lavage with sterile Roswell Park Memorial Institute (RPMI) 1640 medium. Macrophages (10^6^ cells/mL) were cultured overnight in RPMI containing 2% fetal calf serum. Non-adherent cells were removed after phosphate-buffered saline (PBS) wash. Macrophages were stimulated with POPMt (0.01–10 mg/mL), either native or denatured by boiling, for 24 h at 37°C in CO_2_ atmosphere. Macrophages viability assessed by trypan blue exclusion at the end of each experiment was always >95%.

### Immunomodulatory Properties of POPMt

Cell-free supernatants from *in vitro* POPMt stimulated macrophages were collected after 24 h and stored at -20°C until analysis. TNF-α, IL-6, IL-12p70, IL-23, IL-10, IL-1b, and MCP-1 levels were measured in supernatants from *in vitro* POPMt stimulated macrophages by enzyme-linked immunosorbent assay, in accordance with manufacturer instructions (R&D Systems).

### Statistical Analysis

Bars graphics were generated with Prism software (GraphPad). All data was expressed as mean and standard deviation. Statistical analyses were performed by ANOVA followed by the Newman-Keuls-Student and Student’s *t*-tests. The significance level was set at *p* < 0.05.

## Results

### Expression and Purification of POPMt

**FIGURE 1:**
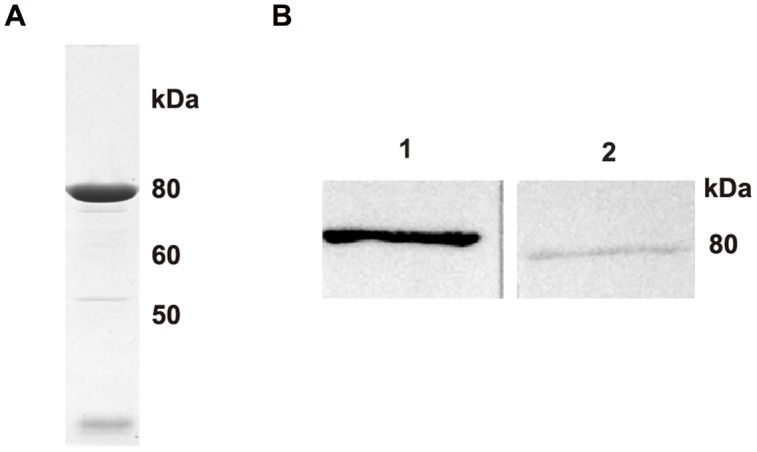
**Purification and analysis of the recombinant and native POPMt. (A)** POPMt was produced in *Escherichia coli* BL21(DE3), purified on nickel-agarose resin and evaluated by comassie-stained 10% SDS-PAGE. **(B)** *Mycobacterium tuberculosis* total protein extract was resolved by 10% SDS PAGE and transferred to nitrocellulose membrane. Blot was probed with anti-POPMt. (1) POPMt purified; (2) *M. tuberculosis* H37Rv total protein extract.

The *popmt* gene from *M. tuberculosis* has an open read frame of 2,022 bp and encodes a protein of 673 amino acids residues with predict molecular mass of 74.40 kDa. POPMt has about 65 and 78% identity compared to *M. smegmatis* and *M. avium* POPs, respectively. On the other hand, POPMt amino acid sequence identity compared to human POP is about 24%. POPMt was expressed in *E. coli* BL21(DE3) as a soluble and active enzyme which allowed us to proceed to its purification (**Figure 1A**) and biochemical characterization. When subjected to intra-dermal immunization, isogenic BALB/c yielded specific anti-POPMt serum capable of recognizing a single band of approximately 75 kDa in the total extract of *M. tuberculosis* proteins. This result confirms that POPMt is immunogenic in mice and is expressed in H37Rv, a *M. tuberculosis* human pathogenic strain (**Figure 1B**).

### Substrate Specificity and Kinetic Parameters of POPMt

Some fluorogenic peptides were used to define the substrate specificity of POPMt (**Table 1**). Among these substrates, the enzyme cleaved specifically at the carboxyl terminus of proline residues, feature similar to other POP, which are known to cleave a Pro-Xaa bond in peptides, where Xaa is not a proline residue (Koida and Walter, 1976). With respect to these substrates, the enzyme was active on *N*-Suc-Gly-Pro-Leu-Gly-Pro-AMC and *N*-Suc-Gly-Pro-AMC but totally inactive toward L-Pro-AMC and Gly-Pro-AMC. The K_M_ and K_cat_/K_M_ values of POPMt toward *N*-Suc-Gly-Pro-Leu-Gly-Pro-AMC were 108 μM and 21.83 mM^-1^ s^-1^, respectively.

**Table 1:** POPMt substrate specificity.

Substrate	Relative activity (%)
Ala-Ala-Phe-AMC	n.a
L-Proline-AMC	n.a
Gly-Pro-AMC	n.a
*N*-Succinyl-Ile-Ala-AMC	1.31
*N*-Succinyl-Leu-Tyr-AMC	n.a
*N*-Succinyl-Leu-Leu-Val-Tyr-AMC	n.a
*L*-Leucine-AMC	n.a
*N*-Succinyl-Gly-Pro-AMC	48
*N*-Succinyl-Gly-Pro-Leu-Gly-Pro-AMC	100

*n.a, no activity. Assays were performed by incubating 300 ng of POPMt with 20 μM of each substrate. Substrate hydrolysis was recorded up to 20 min*.

### Effects of NaCl and DTT on POPMt Catalysis

**FIGURE 2:**
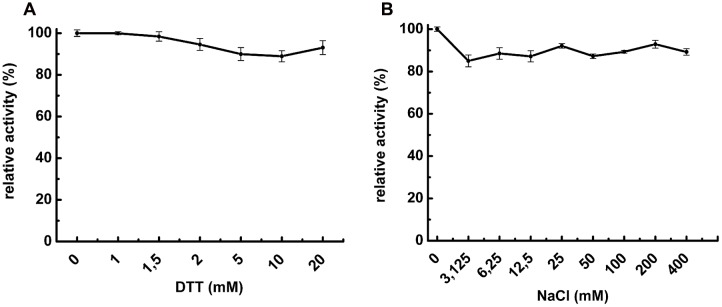
**Effects of additives on POPMt enzymatic activity**. The substrate *N*-Suc-Gly-Pro-Leu-Gly-Pro-AMC was hydrolyzed by POPMt in the presence of **(A)** 0–20 mM DTT; **(B)** 0–400 mM NaCl. Results are expressed as the percent activity relative to the maximum value obtained at each condition.

Thiol-reacting reagents and salts are known to improve POP enzymatic activity (Usuki et al., 2009). Based on that, NaCl and DTT were added into buffer reaction to determine their influence on POPMt enzymatic activity. As shown in **Figure 2A**, up to 20 mM DTT did not affect the enzyme activity. As for DTT, increasing concentrations of NaCl (up to 400 mM) did not alter POPMt enzymatic activity toward *N*-Suc-Gly-Pro-Leu-Gly-Pro-AMC (**Figure 2B**).

### Influence of pH and Temperature on POPMt Structure and Enzymatic Activity

**FIGURE 3:**
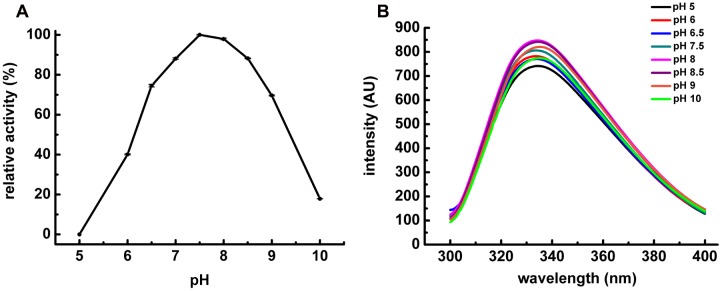
**pH influence on POPMt enzymatic activity and POPMt tertiary structure. (A)** pH optimum enzymatic activity of POPMt was assayed in AMT buffer against *N*-Suc-Gly-Pro-Leu-Gly-Pro-AMC. Results are expressed as the percent activity relative to the maximum value obtained at each condition. **(B)** Intrinsic spectra were recorded at 25°C from 305 to 400 nm using excitation wavelength of 295 nm at different pHs.

**FIGURE 4:**
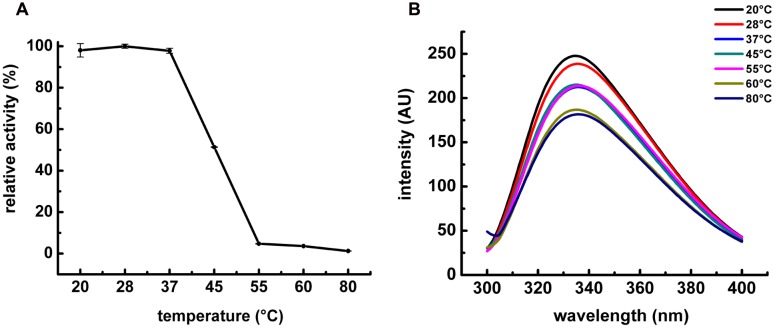
**Influence of temperature on POPMt enzymatic activity and POPMt tertiary structure. (A)** POPMt was incubated with substrate at different temperatures for 20 min and the release of AMC was quantified as described in Section “Materials and Methods.” Results are expressed as the percent activity relative to the maximum value obtained at each condition. **(B)** Intrinsic spectra were recorded from 305 to 400 nm using excitation wavelength of 295 nm at different temperatures.

The characterization of POPMt revealed that the enzyme had a strong dependence on slightly alkaline pH 7.0–8.5. It was most stable at pH 7.5 and retained more than 90% of the residual activity at pH 8.0 and 8.5 (**Figure 3A**). We also analyzed POPMt tertiary structure changes under different pHs by fluorescence spectroscopy (**Figure 3B**). It is possible to observe changes in emission spectra of tryptophan in response to protein conformational transitions, subunit association, substrate binding or denaturation, all of which can affect the local environment surrounding the tryptophan indole ring (Lakowicz, 2004). POPMt contains 20 tryptophan residues and, as shown in **Figure 4B**, the emission maximum was 335 nm, indicating that most POPMt tryptophans are partially buried in the protein. The shape of the enzyme intrinsic fluorescence spectra did not change from pH 7.5 to 8.5 and a limited red-shift happened at pH 9.0 and 10.0.

The effect of temperature on the enzymatic activity of POPMt was examined over a range of 20–80°C at pH 7.5. The enzyme showed maximal enzymatic activity between 20 and 37°C. While at 45°C, the enzyme activity decreased nearly 50% (**Figure 4A**). As for pH analysis, we also applied the tryptophan residue fluorescence as a reporter for structural changes under different temperatures and, according to all the adjusted spectra, temperatures up to 60°C did not alter tryptophan emission pattern (**Figure 4B**).

### Specificity of POPMt Enzymatic Activity Inhibition

**Table 2** summarizes the effects of protease inhibitors on the enzymatic activity of POP from *M. tuberculosis*. Although POPMt is a serine protease, its activity was partially inhibited by AEBSF and TLCK, 33 and 35%, respectively. On the other hand, TPCK, a chymotrypsin-like protease inhibitor, inhibited 66% of the enzyme activity. Leupeptin, E64, pepstatin A and metalloproteinase inhibitors such as *o*-phenanthroline, bestatin and EDTA had no effect on the enzyme activity. The canonical POP inhibitor Z-Pro-Prolinal (Wilk and Orlowski, 1983; Yoshimoto et al., 1987) was also assayed on POPMt, showing a *Ki* value of 16.87 nM. Z-Pro-Prolinal was a less efficient inhibitor when compared to mouse and human POPs with *Ki* value of 0.35 and 0.50 nM, respectively (Bakker et al., 1990).

**Table 2:** Inhibition of POPMt by protease inhibitors.

Inhibitor	Concentration (mM)	Inhibition (%)
AEBSF	1	33
Bestatin	0.1	n.i
E-64	0.1	n.i
EDTA	1	n.i
Leupeptin	0.1	n.i
Pepstatin A	0.1	n.i
Phenanthroline	0.1	n.i
TLCK	0.1	35
TPCK	0.1	66

*n.i, no inhibition. Assays were performed by incubating 300 ng of POPMt with the inhibitor for 15 min, before the addition of 20 μM of substrate. Substrate hydrolysis was recorded up to 20 min*.

### POPMt Homology Modeling and Molecular Dynamics Simulations

Concerning the homology modeling, the top ranked homolog structure returned by BLAST was that of *M. xanthus* (PDB ID 2BKL), with a query coverage of 64 and 37% identity. As we could not obtain higher query coverage, we opted to perform MDs simulations to both refine and validate our model. We predicted that an equilibrium MDs simulation would allow a relaxation of the macromolecule, in which the POPMt features would be adopted instead of those from *M. xanthus* enzyme.

**FIGURE 5:**
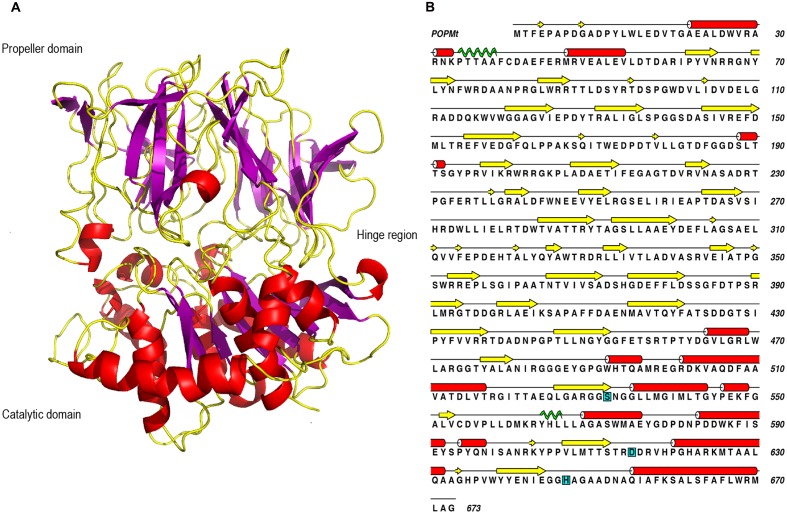
**Sequence and three dimensional structure of POPMt. (A)** POPMt tertiary structure showing α/β hydrolase catalytic domain, β-propeller domain and the hinge region between the two domains. **(B)** The amino acid sequence of POPMt with the assigned secondary structure: coil (black line), β sheet (yellow), α helix (red), Pi helix (green). Catalytic site is highlighted in cyan.

The POPMt macromolecular structure is divided in two domains with a catalytic α/β hydrolase domain and a β-propeller domain (**Figure 5A**). The propeller domain is based on a radially arranged seven-fold repeat of four stranded antiparallel β sheets. In the case of POPs, this domain is considered to be of the “open-velcro” topology, where first and seventh blades are connected only through hydrophobic interactions, although their primary sequences diverge (Kaushik and Sowdhamini, 2011). The POPMt catalytic triad residues (Ser 532, Asp 615, e His 647) position is conserved like in other POPs and it is localized at the interface of the catalytic and propeller domains (**Figure 5B**) (Rea and Fülöp, 2006). The amino acid sequence of POPMt with the assigned secondary structure comprising coil, β sheet, α helix and Pi helix is shown in **Figure 5B**.

**FIGURE 6:**
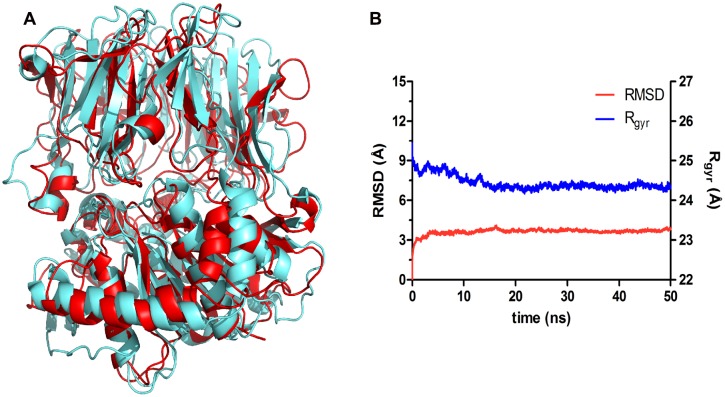
**POPMt 3D model comparison and stability during the molecular dynamics simulation. (A)** Superposition of the POPMt 3D model after MD simulations (cyan) and the POPMx crystallographic structure (red). **(B)** Root-mean square deviation analysis of the trajectory frames (RMSD, red line, left y axis) indicates that the model achieved structural stability after 5 ns of simulation. The decrease of the radius of gyration over simulation time (Rgyr, blue line, right y axis) corroborates the stability hypothesis, suggesting that the protein has become more compact at the end of the simulation.

The superposition of the predicted and template structures (**Figure 6A**) shows an average RMSD of 4.86 Å (**Figure 6B**, red line). This might be explained by the fact that the *M. xanthus* POP template is a crystal structure, hardened due to the compression inherent in the crystallization process, which results in the inability of structure accommodation and its respective adaptation to the environment. Structures subjected to MDs simulations are expected to exhibit RMSD changes, as they go through a process of relaxation and adjustments, always driven by biochemical and structural restrictions.

Albeit resulting in conformational changes when compared to the initial structure, as observed by the RMSD shift, the POPMt relaxation process led the structure to a smaller radius of gyration (**Figure 6B**, blue line), indicating the contraction of the system. This can be interpreted as a sign of conformational stability, as during protein unfolding we would expect the opposite, and may be explained due to the increasing number of intramolecular hydrogen bonds during the simulation (**Supplementary Figure S1**). This kind of interactions cooperates to reduce the volume of the system and thus increase the degree of compactness of the protein.

Another sign of compactness is the solvent accessible area (**Supplementary Figure S1**), which corresponds to the area of solvent removed due to the relationship of the protein with itself. By folding, the protein contracts, resulting in the reduction of the solvent accessible area, decreasing the protein volume characterized by the separation of hydrophobic and hydrophilic portions.

Data extracted from this MD trajectory will be applied to identify clusters of conformational families as recently accomplished to the Dengue virus NS3 protease (de Almeida et al., 2013), which will be used in ensemble docking campaigns aiming the identification of new drug-like hits against POPMt.

### POPMt Induces Proinflammatory Cytokine Production *In vitro*

**FIGURE 7:**
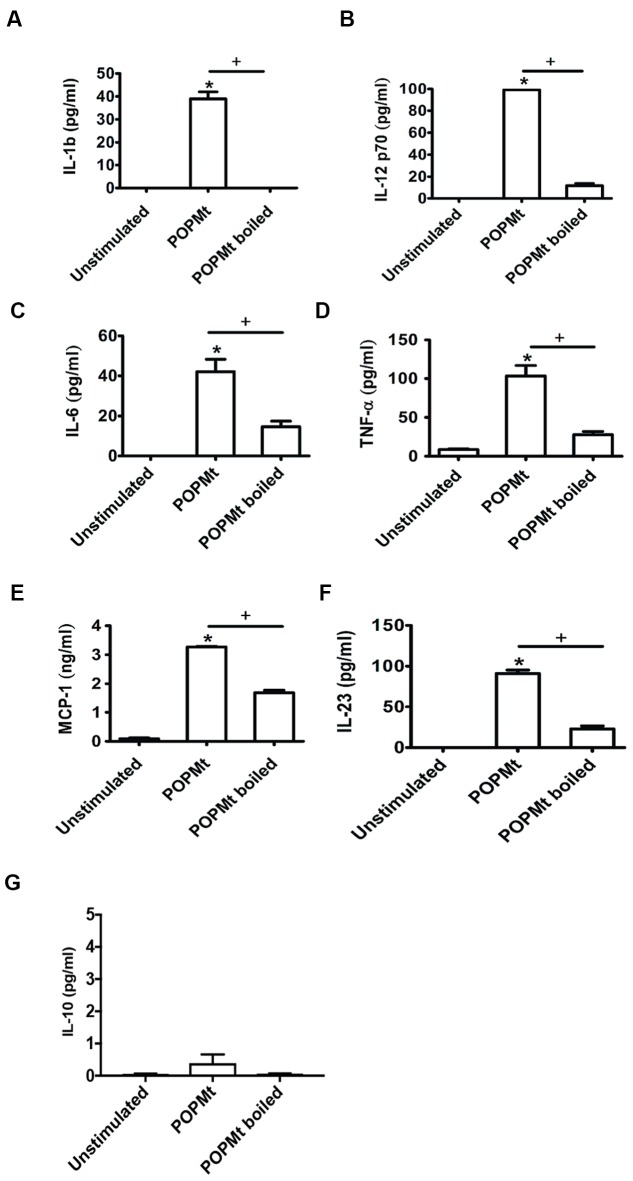
**Immunomodulation triggered by POPMt**. Peritonial macrophages were stimulated with either intact or boiled POPMt for 24 h *in vitro*. Levels of **(A)** IL-1b, **(B)** IL-12p70, **(C)** IL-6, **(D)** TNF-α, **(E)** MCP-1, **(F)** IL-23, and **(G)** IL-10 were analyzed from the supernatant cultured. Statistical significances (*p* < 0.05) between intact and boiled enzymes are represented by the symbol +. Unstimulated versus intact enzyme are represented by the symbol ^∗^.

To investigate the immunomodulatory properties of POPMt in triggering immune responses *in vitro*, peritonial macrophages were stimulated with purified POPMt for 24 h. POPMt significantly triggered production of the proinflammatory cytokines TNF, IL-12p70, IL-6, IL-23 and IL-1b, as well as chemokine MCP-1 after 24 h (**Figures 7A–F**). However, POPMt failed to trigger the anti-inflammatory and immunosuppression cytokine IL-10 (**Figure 7G**). In addition, all analyzed cytokines and chemokine secretion induced by POPMt were dependent on the enzyme intact structure since cytokine and chemokine production induced by boiled POPMt were greatly reduced.

## Discussion

Here we report, for the first time, the identification, purification, biochemical characterization and some immunomodulatory features of a POP from *M. tuberculosis.* POPs have already been described in bacteria like *Flavobacterium meningosepticum* (Yoshimoto et al., 1980, 1991), *Sphingomonas capsulate* (Kabashima et al., 1998), *Aeromonas hydrophila* (Kanatani et al., 1993), and *M. xanthus* (Shan et al., 2004).

Enzymatic tests were performed using *N*-Suc-Gly-Pro-Leu-Gly-Pro-AMC as substrate since it was hydrolyzed more efficiently than the others tested. This result was also observed for POP from *T. cruzi* and *T. brucei* (Santana et al., 1997; Bastos et al., 2010). The additives used in this study (DTT and NaCl) are known to influence the enzymatic activity of POP family, however, such effect was not observed for POPMt. Nevertheless, the insensitivity to thiol-reacting reagents, such as DTT, has been reported for other members of POP family. For example, DTT did not interfere in the enzymatic activity of oligopeptidase B from *T. cruzi* (Motta et al., 2012) neither in POP from *F. meningosepticum* and *Lyophyllum cinerascens* (Yoshimoto et al., 1988, 1991). Oligopeptidase B from *T. brucei* has a Cys residue located at position 256 that was identified as the one responsible for interaction with thiol reagents (Morty et al., 2005). It also has been shown that porcine brain POP is inhibited by pCMB, a cysteine protease inhibitor, possibly by its reaction with Cys_256_, a residue located close to the enzyme substrate binding site (Szeltner et al., 2000). Based on our model, the two Cys residues predicted in POPMt primary sequence (Cys40 and Cys554) are not located near the catalytic site. Moreover, according to our MD studies, the distance between these POPMt Cys residues remained at approximately 17 Å throughout the simulation, such distance is not favorable for formation of a disulfide bridge, which is postulated between 4.4 and 6.8 Å (Richardson, 1981). Both features may explain the insensitivity of POPMt to thiol reagents.

POPMt optimum pH and temperature is at 7.5 and 37°C, respectively. These values are compatible with those already described for other POPs (Yoshimoto et al., 1980; Szwajcer-Dey et al., 1992). Fluorescence spectroscopy has been widely used in structural and functional studies of proteins. The intrinsic fluorescence of the tryptophan residues of proteins is a natural sensitive probe for protein denaturation and can be used in experiments of protein tertiary structure (Lakowicz, 2004). These approaches have already been used to probe into the tertiary structure unfolding of two carbonic anhydrase, Rv3588c and Rv1284, both from *M. tuberculosis* (Mukherjee et al., 2009). The spectra of POPMt tryptophan fluorescence did not alter the maximum emission wavelength in response to changes in pH, suggesting that POPMt preserved a similar degree of structure folding throughout the experiment. Notably, POPMt unfolding was minimum when temperature increased. The maximum emission intensity for POPMt decreased following temperature increasing and, on the other hand, the maximum emission wavelength did not (red)shift, suggesting, as for pH, a similar degree of structure folding. Even though it appears that POPMt tertiary structure is stable regarding to pH and temperature changes, it is necessary to take into account that this enzyme contains 20 tryptophan residues, which could influence their utilization as reporter groups in studying protein (un)folding (Pokalsky et al., 1995).

Extracellular matrix constituent destruction is critical to the success of *M. tuberculosis* infection, but the essential mechanisms of this destruction remain poorly understood. It has been mainly proposed that host matrix metalloproteinases (MMPs) play a central role in the event, owing to their unique ability to degrade fibrillar collagens and other matrix components (Elkington et al., 2011). Besides tissue damage, collagen fragmentation may generate active peptides named matrikines, which have been implicated in immune response by altering cellular migration and chemotaxis (Wells et al., 2015). Emerging evidences has suggested that POPs could participate in the inflammatory response through the modulation of active peptides (Gaggar et al., 2010; Tenorio-Laranga et al., 2013, 2015). For instance, in concert with MMPs, POP is responsible for generation of one type of matrikine, the tripeptide PGP (proline-glycine-proline), a neutrophil chemoattractant that has been implicated in inflammation and disorders of the respiratory system (O’Reilly et al., 2009).

Inflammatory cytokines such as IFNγ, mainly secreted by T lymphocytes, could activate macrophage antimicrobial mechanisms against *M. tuberculosis*. Moreover, TNFα is a crucial proinflammatory cytokine for *M. tuberculosis* control in humans and in experimental animals (Solovic et al., 2010), by contributing to activation of macrophages for killing of intracellular mycobacteria and to modulate apoptosis of infected cells (Balcewicz-Sablinska et al., 1998; Clay et al., 2008). Unlike TNFα, IL-6 is critical to resistance against *M. tuberculosis*, but it is dispensable for the control of mycobacterial growth after low-dose aerosol-delivered infection (Sodenkamp et al., 2012). Despite its importance in mediating inflammation, IL-6 is not as essential as TNFα for antimycobacterial effector mechanisms (Ladel et al., 1997; Saunders et al., 2000; Nagabhushanam et al., 2003; Martinez et al., 2013). Another important macrophage produced proinflammatory cytokine essencial to *M. tuberculosis* killing is IL-1β, which can also regulate Th17 cytokines secretion. Mice that lack IL-1β or its receptor are highly susceptible to *M. tuberculosis* infection, and IL-1β directly inhibits *M. tuberculosis* intracellular growth (Jayaraman et al., 2010, 2013; Mayer-Barber et al., 2010; Qiu et al., 2012; Sada-Ovalle et al., 2012). Here, we have showed that POPMt is capable of immunomodulating murine macrophages by inducing the secretion of the proinflammatory Th1 cytokines TNFα, IL-1β, IL-6, IL-12, as well as the Th17 IL-23 in murine macrophages, indicating that POPMt could significantly contribute to TB immunopathology.

*Mycobacterium tuberculosis* infection is also characterized by induction of elevated levels of a variety of chemokines, such as CXCL-8, MCP-1, MCP-3, MCP-5, RANTES, MIP1-α, MIP1-β, MIP-2 and IP-10 and their receptors (Orme and Cooper, 1999; Juffermans et al., 2000; Mihret et al., 2013). Our data demonstrated that POPMt induced high levels of MCP-1 secretion, suggesting that it could be critical in monocyte/macrophage recruitment process. Beyond monocyte/macrophage recruitment, these inflammatory chemokine recruit additional cells from the blood compartment (Gerszten et al., 1999) and from other areas of the lung (Sertl et al., 1986; Holt and Schon-Hegrad, 1987) to amplify the inflammatory response. In addition, MCP-1 is associated with the immune response to *M. tuberculosis* infection, as MCP-1 deficient mice had early and persistent defects on macrophage recruitment to the lungs and a reduced number of macrophage and dendritc cells in mediastinal linfonodes (Peters et al., 2001, 2004).

We have provided the first biochemical and structural analysis of POPMt together with some *in vitro* murine macrophages immunomodulatory response elicited by the intact protease. More studies must be carried out to determining if native POMt has a role in *M. tuberculosis* infection. If ratified, POPMt could be proposed as new target for the development of anti-mycobacteria drugs.

## Ethics Statement

This study was carried out in accordance with the recommendations of Comissão de Ética no Uso de Animais da Universidade de Brasília. The protocol was approved by Comissão de Ética no Uso de Animais da Universidade de Brasília.

## Author Contributions

Conceived and designed the experiments: BP, FM, DN, KM, IB, and JS. Performed the experiments: BP, FM, AC, DN, KM, and FV Analyzed data: BP, FM, DN, HdA, KM, and IB. Contributed reagents/materials/analysis tools: DN, AA, IB, and JS. Wrote the paper: BP, FM, AC, DN, HdA, KM, IB, and JS.

## Conflict of Interest Statement

The authors declare that the research was conducted in the absence of any commercial or financial relationships that could be construed as a potential conflict of interest.

## Supplementary Material

FIGURE 1 **Plots from molecular dynamics of POPMt. (A)** Radius of gyration versus time plot. **(B)** Hydrogen bonds versus time plot. **(C**) Solvent accessible area versus time plot.
