# Supplementary figures and images for: Mycobacterium tuberculosis Prolyl Oligopeptidase Induces In vitro Secretion of Proinflammatory Cytokines by Peritoneal Macrophages
Source: Front Microbiol. 2017 Feb 7;8:155. doi: 10.3389/fmicb.2017.00155 (PMC5293833; doi:10.3389/fmicb.2017.00155)

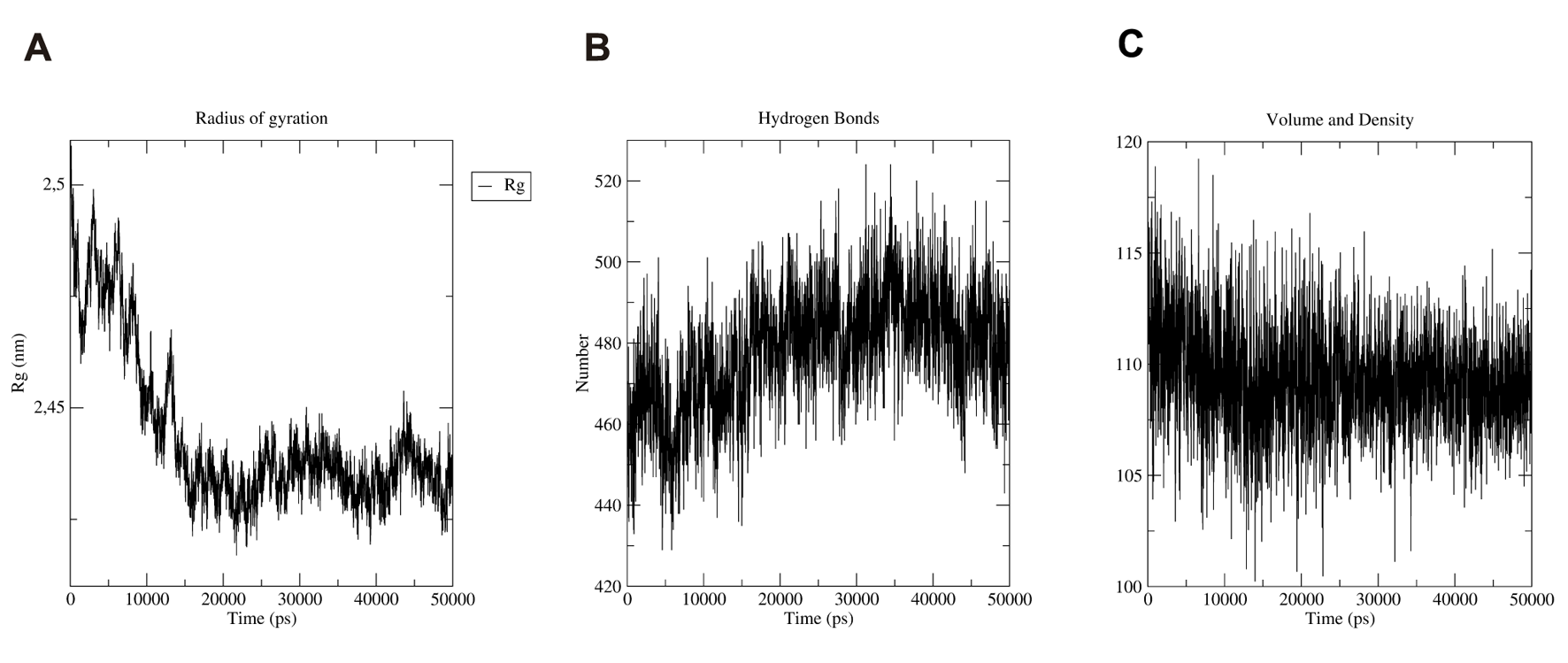

Supplement: FIGURE 1 — Plots from molecular dynamics of POPMt. (A) Radius of gyration versus time plot. (B) Hydrogen bonds versus time plot. (C) Solvent accessible area versus time plot. [file Image_1.TIF]
